# Diagnostic Value and Metabolic Association of Serum Clusterin in Women with Polycystic Ovary Syndrome

**DOI:** 10.3390/diagnostics16010167

**Published:** 2026-01-05

**Authors:** Dilara Sarıkaya Kurt, Recep Taha Ağaoğlu, Mehmet Ferdi Kıncı, Tuğçe Sırma, Ahmet Kurt, Ramazan Erda Pay, İsmail Burak Gültekin, Hüseyin Levent Keskin, Sezin Ertürk Aksakal

**Affiliations:** 1 Department of Obstetrics and Gynecology, Etlik City Hospital, Ankara 06170, Türkiye; mflkurt@gmail.com (A.K.); drramazanpay@gmail.com (R.E.P.); burakgultekin@yahoo.com (İ.B.G.); hlkeskin@yahoo.com (H.L.K.); drsezert@gmail.com (S.E.A.); 2 Department of Perinatology, Etlik City Hospital, Ankara 06170, Türkiye; tahaagaoglu@hotmail.com; 3 Department of Obstetrics and Gynecology, İzmir City Hospital, İzmir 35400, Türkiye; drferdikinci@gmail.com; 4 Department of Gynecological Oncology Surgery, İskenderun State Hospital, Hatay 31200, Türkiye; drtugcesirma@hotmail.com; 5 Department of Obstetrics and Gynecology, School of Medicine, Ufuk University, Ankara 06510, Türkiye

**Keywords:** polycystic ovary syndrome, clusterin, oxidative stress, biomarker

## Abstract

**Background:** Polycystic ovary syndrome (PCOS) is a common endocrine and metabolic disorder characterized by hyperandrogenism, ovulatory dysfunction, and an increased prevalence of metabolic syndrome. Clusterin (CLU), a chaperone protein induced by cellular stress and known to play roles in inflammation, oxidative stress, and lipid metabolism, may be associated with the metabolic abnormalities observed in patients with PCOS. The purpose of this current study is to investigate serum CLU levels and their link with endocrine, biochemical, and metabolic parameters, such as metabolic syndrome, among women with PCOS. **Methods:** This cross-sectional study included 40 women aged 18–30 with PCOS diagnosed according to the Rotterdam criteria and 40 age- and BMI-matched healthy controls. Demographic data, Ferriman–Gallwey scores, hormonal and metabolic parameters (including TSH, prolactin, 17-OH progesterone, total testosterone, insulin, AMH, HOMA-IR, and serum CLU levels), and ultrasonographic ovarian morphology were assessed. Statistical analyses, including ROC and logistic regression, were performed. **Results:** Women with PCOS had higher follicle counts, Ferriman–Gallwey scores, LH/FSH ratios, fasting insulin levels, HOMA-IR, triglycerides, and systolic blood pressure than controls, whereas menstrual cycle frequency and HDL levels were lower (all *p* < 0.05). Serum CLU concentrations were markedly higher in the PCOS cohort. In the PCOS population, CLU showed positive relationships with the Ferriman–Gallwey score, fasting glucose, fasting insulin, HOMA-IR, and triglycerides, and a negative correlation with HDL. CLU levels were significantly higher in women with metabolic syndrome in the PCOS cohort compared to those without. In logistic regression analysis, CLU, AMH, and the LH/FSH ratio emerged as independent predictors of PCOS. Furthermore, CLU remained an independent predictor of metabolic syndrome in the PCOS cohort. In ROC analysis, CLU demonstrated strong diagnostic efficacy in detecting both PCOS (AUC = 0.834) and metabolic syndrome in patients with PCOS (AUC = 0.804). **Conclusions:** Our results show that serum CLU is higher in women with PCOS and is associated with the clinical and metabolic features peculiar to patients with PCOS. CLU was found to distinguish between patients with PCOS and healthy women and demonstrated a strong association with the presence of metabolic syndrome within the PCOS group. Overall, these findings suggest that CLU may be a valuable auxiliary biomarker for detecting women with PCOS at risk for metabolic disturbances.

## 1. Introduction

Polycystic ovary syndrome (PCOS) is one of the most frequent endocrine disorders affecting women of reproductive age, with an estimated prevalence of 6% to 15% [1]. This disease is heterogeneous, presenting with endocrine disturbances such as menstrual irregularities, infertility, and hyperandrogenism, alongside significant metabolic complications [2,3]. PCOS can occur at any point during the reproductive years due to disrupted interactions among the central nervous system, pituitary gland, adrenal glands, and extraglandular tissues [4]. The syndrome is associated with long-term risks such as insulin resistance, type 2 diabetes mellitus, metabolic syndrome, dyslipidemia, and cardiovascular disease, highlighting its systemic nature.

The pathophysiology of PCOS is multifaceted and is considered to develop from interactions among genetic predisposition, environmental factors, and endocrine system alterations [5,6]. Insulin resistance and hyperinsulinemia are fundamental pathophysiological processes. Hyperinsulinemia stimulates androgen production from the ovaries and is recognized as a contributing cause to ovulatory dysfunctions. In the other direction, chronic inflammation, oxidative stress, and variations in adipokine and hepatokine concentrations have been identified as key contributors to the pathophysiology of the metabolic and hormonal imbalances observed in patients with PCOS [7,8]. In conclusion, considering the close connection between metabolic and hormonal imbalances, the pathophysiological processes highlight the need to develop effective biomarkers for PCOS. Despite the impact of comprehensive laboratory tests and ultrasonographic evaluation on clinical perception and decision-making, laboratory tests are currently used to confirm the diagnosis and to rule out other causes [9,10].

Clusterin (apolipoprotein-J, CLU) is a stress-responsive, multifunctional glycoprotein produced by several tissues, including the liver, adipose tissue, gonads, vascular endothelium, and various epithelial cells, before entering the circulation [11]. As an extracellular chaperone, it helps stabilize misfolded or damaged proteins and limits inflammation, in part by inhibiting the complement cascade [11]. CLU is also involved in immune regulation, lipid handling, and cellular stress responses. A considerable portion of circulating CLU is bound to HDL particles, where it contributes to their antioxidant and antiatherogenic functions. When CLU dissociates from HDL, these protective properties are reduced, reflecting alterations in lipoprotein quality and function [11,12]. Research indicates that CLU expression and circulating levels increase with obesity and visceral adiposity, correlating with chronic low-grade inflammation, high-sensitivity C-reactive protein (hs-CRP), retinol-binding protein-4, and other proinflammatory markers [13]. Moreover, adipocyte-derived CLU has been shown to inhibit insulin signaling in the liver, thereby increasing gluconeogenesis, and may function as both a marker and a facilitator of insulin resistance [14]. Decreased CLU levels in HDL have been documented to be significantly associated with insulin resistance, obesity, and atherogenic dyslipidemia [12]. Given the central obesity, hyperinsulinemia, chronic low-grade inflammation, and atherogenic lipid profile associated with PCOS, CLU is significant as a possible biomarker for metabolic stress and insulin resistance in this condition [15].

Therefore, the present study aimed to evaluate serum clusterin levels in women with polycystic ovary syndrome and to investigate its associations with metabolic and hormonal parameters, as well as its diagnostic value and metabolic relevance within the PCOS population.

## 2. Materials and Methods

### 2.1. Study Setting and Design

This cross-sectional study was conducted at Etlik City Hospital (Ankara, Türkiye) between December 2024 and February 2025, following approval from the ethics committee (Approval No: AEŞH-BADEK-2024-1161; Date: 11 December 2024). This study was conducted in accordance with the principles of the Declaration of Helsinki, and all participants provided written informed consent before enrollment.

### 2.2. Participants

A total of 40 women aged 18–30 years, diagnosed with PCOS and not on regular medication, were included in the study. The control group consisted of 40 healthy women with regular menstrual cycles, matched by age and body mass index (BMI), for whom routine blood tests had been requested. PCOS was diagnosed based on the Rotterdam criteria, which require the presence of at least two of the following three features [2]: (i) clinical and/or biochemical signs of hyperandrogenism; (ii) oligomenorrhea or amenorrhea; (iii) polycystic ovarian morphology on ultrasound. In the present study, the majority of patients fulfilled the criteria for the classic PCOS phenotype, characterized predominantly by hyperandrogenism and ovulatory dysfunction, thereby representing an enriched PCOS population. Exclusion criteria included liver or kidney disease, diabetes mellitus, heart failure, acute infection, hormone or insulin-sensitizing drug use within the last three months, and conditions causing hyperandrogenemia or ovulatory dysfunction (e.g., non-classical congenital adrenal hyperplasia, Cushing’s syndrome, androgen-secreting tumors of the ovary or adrenal gland, thyroid disorders, hyperprolactinemia, hypogonadism, functional hypothalamic amenorrhea, and premature ovarian failure).

### 2.3. Data Collection and Measurements

For all participants, the following data were collected: age, height, weight, BMI, Ferriman–Gallwey score, and laboratory values including TSH, prolactin, 17-OH progesterone, total testosterone, fasting blood glucose, fasting insulin, HOMA-IR, hemogram parameters, HDL, LDL, triglycerides, estradiol, AMH, FSH, LH, the LH/FSH ratio, sex hormone-binding globulin (SHBG), and CLU levels. Free Androgen Index (FAI) was calculated using the following formula: (total testosterone/sex hormone-binding globulin (SHBG)) × 100. HOMA-IR was calculated using the following formula: (Fasting blood glucose (mg/dL) × Fasting insulin (µU/mL))/405. Ultrasound evaluations of the uterus and ovaries were performed via transvaginal or abdominal ultrasonography. During the first 7 days of the menstrual cycle, five cc of blood was collected for hormonal, insulin, and FSH testing. After appropriate centrifugation, samples were stored at −80 °C until CLU analysis.

### 2.4. Definitions

Oligo-ovulation was defined as having fewer than eight spontaneous ovulatory menstrual cycles per year or a menstrual cycle length ≥ 35 days. Anovulation was defined as the complete absence of ovulatory menstrual cycles within the assessment period.

Metabolic syndrome (MS) was defined according to the National Cholesterol Education Program Adult Treatment Panel III (NCEP ATP III) criteria [16], with a minor modification in which BMI was used instead of waist circumference. Because waist circumference measurements were unavailable for all participants, a BMI ≥ 30 kg/m^2^ was used as a surrogate marker of central obesity. Accordingly, MS was diagnosed when at least three of the following criteria were met:

BMI ≥ 30 kg/m^2^;

Triglyceride level ≥ 150 mg/dL;

HDL cholesterol < 50 mg/dL;

Blood pressure ≥ 135/85 mmHg;

Fasting plasma glucose ≥ 100 mg/dL.

### 2.5. Measurement of Serum CLU Levels

Serum CLU concentrations were measured using a commercial enzyme-linked immunosorbent assay (ELISA) kit (USCN, Wuhan USCN Business Co., Ltd., Wuhan, China; Catalog No.: USEB180Hu) based on the double-antibody sandwich principle. According to the manufacturer’s specifications, the assay detection range was 12.5–800 ng/mL, with a minimum detectable concentration of <5.1 ng/mL. The intra-assay coefficient of variation (CV) was reported to be <10%, and the inter-assay CV was <12%. Samples were thawed only once to avoid protein degradation.

### 2.6. Study Objectives

The primary objective of this study was to compare serum clusterin (CLU) levels between women with polycystic ovary syndrome (PCOS) and age- and body mass index-matched healthy controls. The secondary objectives were to examine the relationships between CLU levels and hormonal, metabolic, and clinical parameters, and to evaluate the diagnostic performance of CLU for identifying PCOS and its association with CLU levels and metabolic syndrome within the PCOS cohort.

### 2.7. Sample Size Calculation

The sample size was determined a priori using G*Power 3.1.9.7 (University of Düsseldorf, Düsseldorf, Germany). The calculation was based on the study by Chen et al. [4], who reported significantly higher CLU levels in women with PCOS compared to healthy controls. Using the difference in CLU levels between groups reported in that study, the effect size (Cohen’s d) was calculated as 2.75. With a two-tailed test, an alpha level of 0.05, and a power (1–β) of 0.99, the minimum required sample size was 14 participants in total (7 in each group). However, to increase statistical robustness, improve generalizability, and allow for potential data loss, we included a substantially larger sample. Therefore, the present study recruited 40 women with PCOS and 40 age- and BMI-matched healthy controls (total *n* = 80).

### 2.8. Statistical Analysis

SPSS 31.0 was used to perform statistical analyses in the study. Descriptive statistics for categorical variables are presented as frequencies and percentages. The Shapiro–Wilk test was used to assess the normality of continuous variables. Normally distributed variables were analyzed using Student’s *t*-test and are reported as mean ± standard deviation (SD), whereas non-normally distributed variables were analyzed using the Mann–Whitney U test and are reported as median (25th–75th percentile). Spearman and Pearson correlation analyses were used to evaluate the relationships between CLU levels and other continuous parameters. Receiver operating characteristic (ROC) curve analysis was used to evaluate the diagnostic performance and discriminative ability of serum CLU levels by estimating sensitivity, specificity, and optimal cut-off values. Binary logistic regression analysis was performed to determine whether CLU was independently associated with PCOS after adjustment for potential confounding variables. In addition, ROC analysis and binary logistic regression were also applied within the PCOS group to assess both the predictive performance and independent association of CLU and other parameters with metabolic syndrome. A *p*-value of <0.05 was considered statistically significant.

## 3. Results

A total of 64 women with PCOS were initially assessed for eligibility during the study period. Among them, 24 participants were excluded based on the predefined exclusion criteria, including diabetes mellitus (*n* = 6), thyroid disorders (*n* = 5), recent use of hormonal or insulin-sensitizing medications (*n* = 7), and other causes of hyperandrogenism or ovulatory dysfunction (*n* = 6). After these exclusions, 40 women with PCOS and 40 age- and body mass index–matched healthy controls were included in the final analysis.

The study included 40 women with PCOS and 40 age- and BMI-matched healthy controls who met the inclusion criteria. As shown in Table 1, gravidity and parity were significantly lower in the PCOS group than in the control group (both *p* < 0.001). The follicle count was markedly higher in the PCOS group (*p* < 0.001). In addition, the number of menstrual cycles per year was significantly lower in the PCOS group than in controls (*p* < 0.001). The Ferriman–Gallwey score was also significantly higher among women with PCOS (10 (9–12)) than in the control group (5 (4–8)) (*p* < 0.001).

The comparison of hematological, biochemical, and coagulation parameters between the PCOS and control groups is given in Table 2. Fasting glucose levels were significantly higher in the PCOS group compared with the control group (*p* = 0.032). The PCOS group also had significantly higher fasting insulin levels (*p* < 0.001) and HOMA-IR scores (*p* = 0.002). Serum LH levels and the LH/FSH ratio were significantly higher in the PCOS group compared with controls (both *p* < 0.001), while FSH levels were similar between groups (*p* = 0.256). Total testosterone levels and FAI were significantly higher in women with PCOS (*p* < 0.001 and *p* = 0.002, respectively). In addition, AMH levels were markedly higher in the PCOS group (*p* < 0.001). Serum CLU levels were significantly higher in the PCOS group compared with the control group (24.814 ± 2.875 vs. 21.386 ± 1.975 µg/mL, *p* < 0.001), with an effect size of Cohen’s d = 1.422.

The relationships between CLU and other factors in the PCOS cohort are summarized in Table 3. CLU levels demonstrated significant positive correlations with Ferriman–Gallwey score (r = 0.456, *p* < 0.001), fasting insulin (r = 0.619, *p* < 0.001), and HOMA-IR score (r = 0.597, *p* < 0.001). CLU was also positively correlated with fasting plasma glucose (r = 0.457, *p* < 0.001). A moderate statistically significant positive correlation was observed between CLU and TG (r = 0.346, *p* = 0.029). However, CLU was negatively correlated with HDL (r = −0.419, *p* = 0.007).

Among women with PCOS, no significant differences were found between the metabolic syndrome (MS) and non-MS groups in terms of age, follicle count, and menstrual cycle frequency (*p* > 0.05). Ferriman–Gallwey scores were significantly higher in the MS group (*p* = 0.017). As expected, HOMA-IR score was markedly higher in women with MS (4.50 ± 2.02 vs. 2.64 ± 1.06, *p* = 0.002). No significant differences were observed in gonadotropins, estradiol, TSH, 17-OH progesterone, prolactin, total testosterone, DHEA-S, SHBG, AMH, or FAI between the groups (all *p* > 0.05). CLU levels were significantly higher in the MS group compared with non-MS patients (*p* < 0.001). These results are shown in Table 4.

Table 5 displays the results of the logistic regression analysis for predicting PCOS and MS in the PCOS cohort. In the univariable logistic regression analysis performed in the entire cohort, several parameters were significantly associated with the presence of PCOS, including HOMA-IR (OR: 1.863, 95% CI: 1.247–2.783, *p* = 0.002), LH/FSH ratio (OR: 16.258, *p* < 0.001), total testosterone (OR: 1.076, *p* < 0.001), CLU (OR: 1.807, 95% CI: 1.371–2.380, *p* < 0.001), and AMH (OR: 1.561, 95% CI: 1.268–1.922, *p* < 0.001). Following multivariable analysis adjustment using backward LR regression, only three variables remained independent predictors of PCOS: LH/FSH ratio (aOR: 7.810, 95% CI: 1.436–42.476, *p* = 0.017), CLU (aOR: 1.853, 95% CI: 1.252–2.743, *p* = 0.002), and AMH (aOR: 1.564, 95% CI: 1.202–2.036, *p* < 0.001).

Within the PCOS subgroup, univariable regression identified Ferriman–Gallwey score (OR: 1.769, 95% CI: 1.176–2.663, *p* = 0.006), HOMA-IR (OR: 2.190, *p* = 0.004), and CLU (OR: 1.631, 95% CI: 1.193–2.228, *p* = 0.002) as significant predictors of metabolic syndrome. In multivariable analysis, age (aOR: 1.328, 95% CI: 1.001–1.762, *p* = 0.049), Ferriman–Gallwey score (aOR: 1.816, 95% CI: 1.073–3.075, *p* = 0.026), and CLU (aOR: 1.577, 95% CI: 1.099–2.263, *p* = 0.013) remained independent predictors of metabolic syndrome within the PCOS cohort.

**Figure 1:**
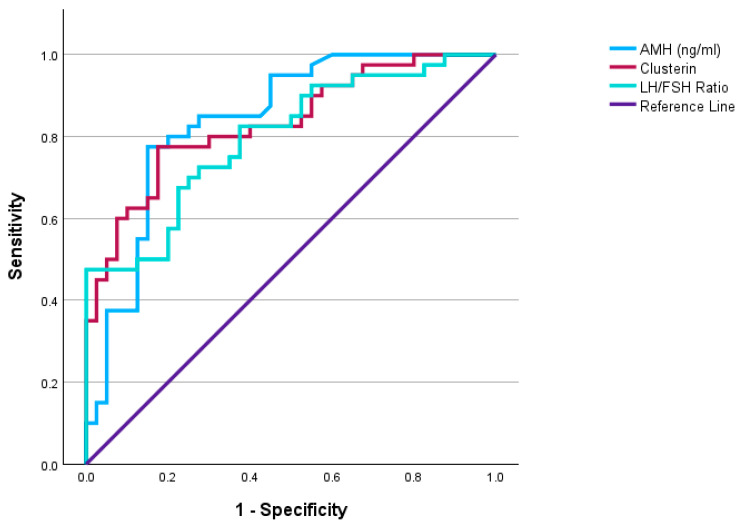
Diagnostic performance of clusterin, AMH, and LH/FSH ratio in identifying PCOS.

Figure 1 and Table 6 present the receiver operating characteristic (ROC) analysis. ROC curve analysis demonstrated that serum CLU had a strong diagnostic performance in distinguishing women with PCOS from healthy controls, with an optimal cut-off value of ≥22.68 µg/mL yielding an AUC of 0.834 (95% CI: 0.746–0.921), 78% sensitivity, and 83% specificity (*p* < 0.001). AMH also showed a high discriminative ability for PCOS (AUC: 0.841, sensitivity 78%, specificity 85%, *p* < 0.001), while the LH/FSH ratio (AUC: 0.798) demonstrated moderate predictive performance.

**Figure 2:**
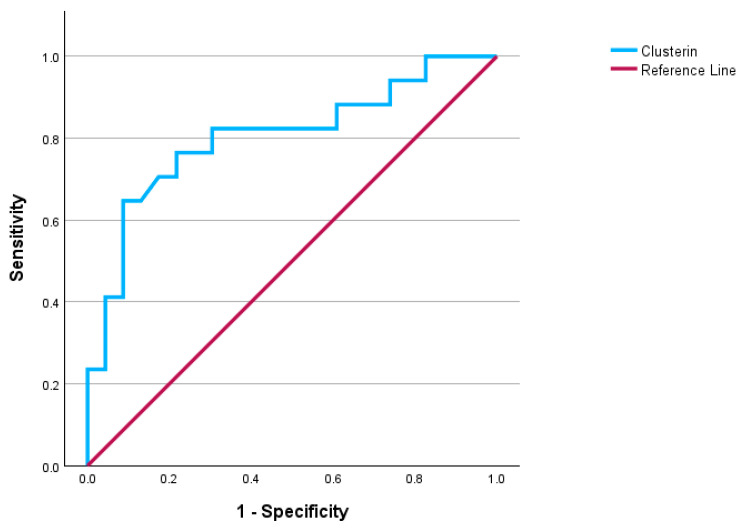
Diagnostic Performance of clusterin in identifying metabolic syndrome among PCOS patients.

Within the PCOS group, CLU remained a significant predictor of metabolic syndrome, with a cut-off value of ≥27.30 µg/mL achieving an AUC of 0.804 (95% CI: 0.659–0.950), 65% sensitivity, and 91% specificity (*p* < 0.001) (Figure 2).

## 4. Discussion

This research showed that serum CLU levels are higher in PCOS patients and are associated with metabolic indicators, including the Ferriman–Gallwey score, fasting glucose, insulin, HOMA-IR score, and HDL levels. In ROC analyses, CLU accurately differentiated PCOS. Multivariable regression analysis confirmed that CLU was an independent predictor of PCOS, along with LH/FSH ratio and AMH. In the PCOS cohort, CLU levels were considerably higher in patients with metabolic syndrome and were identified as independent predictors of the condition.

PCOS is increasingly viewed as a heterogeneous condition encompassing distinct reproductive and metabolic phenotypes, rather than a single uniform disorder [1,3,9]. A considerable percentage of patients demonstrate insulin resistance, hyperinsulinemia, abdominal obesity, and atherogenic dyslipidemia. These metabolic conditions exacerbate hyperandrogenism and markedly heighten long-term cardiometabolic risks [17]. Numerous studies indicate that HOMA-IR levels are significantly enhanced in PCOS compared to healthy women, triglyceride levels are increased, and HDL is substantially diminished [15,18]. Metabolic syndrome is commonly encountered in women with polycystic ovary syndrome [19]. This metabolic setting renders the exploration of novel biomarkers linked to inflammation, insulin resistance, and lipid metabolism disorders therapeutically significant; one molecule of interest in this regard is CLU.

CLU is recognized as contributing to the enhanced protective response during cellular stress, inflammation, and metabolic dysregulation; nevertheless, research indicates that this protein may serve not only as a marker but also as an active participant in these processes [20]. Won and colleagues indicated that circulating CLU levels are significantly correlated with adiposity and inflammatory markers, including BMI, waist circumference, hs-CRP, and RBP-4, and established that CLU is indicative of obesity-related chronic inflammation [13]. Hoofnagle et al. reported lower levels of HDL-associated clusterin with increasing insulin resistance and dyslipidemia, suggesting HDL dysfunction and an atherogenic lipid profile [12]. Bradley et al.’s in vitro and in vivo investigations showed that adipocyte-derived CLU inhibits hepatic insulin signaling, enhances gluconeogenesis, and induces metabolic stress in the liver [20]. Recent evidence indicates that circulating clusterin levels are closely associated with cardiometabolic risk parameters, suggesting that elevated CLU may represent a stress-responsive or compensatory biomarker accompanying metabolic dysregulation, including insulin resistance, rather than a direct causal mediator [11]. The unifying theme throughout these several investigations is that CLU is significantly linked to obesity, inflammation, insulin resistance, and dyslipidemia. The essential pathophysiology components of PCOS overlap with these pathways, making the relationship of CLU with PCOS, particularly the metabolic syndrome phenotype within PCOS in our study, a physiologically anticipated conclusion consistent with the existing literature.

Examining research on CLU in PCOS, Asar et al. [21] found that women with PCOS had significantly higher CLU levels than healthy controls, and that CLU levels were even higher in PCOS patients with metabolic syndrome. Additionally, a weak but substantial positive association between HOMA-IR and insulin and CLU was discovered. Likewise, Chen et al. found higher CLU levels in women with PCOS, independent of BMI, and identified a positive correlation between CLU and HOMA-IR, fasting glucose, and insulin levels [15]. Numan et al. reported in another study that CLU levels were higher in patients with PCOS, demonstrating high sensitivity and specificity for distinguishing PCOS (AUC = 0.944) and a significant correlation with metabolic parameters, including HDL, triglycerides, and total cholesterol [22]. Our data indicate that CLU levels were markedly higher in the PCOS group relative to healthy persons, as well as in patients with metabolic syndrome within the PCOS subgroup. Moreover, in accordance with the literature, CLU had a positive correlation with HOMA-IR, triglycerides, and the Ferriman–Gallwey score, while demonstrating a negative correlation with HDL. The metabolic findings of Bradley et al. indicate that adipose-derived CLU inhibits the insulin signaling pathway and directly contributes to insulin resistance by enhancing gluconeogenesis, thereby substantiating the biological correlation of CLU with PCOS and metabolic syndrome in our study [20].

With these metabolic findings in mind, an important clinical consideration is how CLU differs from other biomarkers commonly used in polycystic ovary syndrome. AMH is a well-established biomarker reflecting follicular excess and ovarian dysfunction and is primarily used to characterize the reproductive phenotype of PCOS. Accordingly, AMH mainly represents ovarian morphology and reproductive features rather than the metabolic or cardiometabolic aspects of the disease. In contrast, widely used metabolic markers typically reflect isolated components of metabolic dysfunction, such as insulin resistance or dyslipidemia. CLU, however, is closely associated with multiple interconnected pathways, including insulin resistance, chronic low-grade inflammation, lipid metabolism, and oxidative stress, thereby representing an integrated metabolic response. From this perspective, CLU should be regarded as a complementary biomarker rather than an alternative to existing hormonal or metabolic markers, providing additional insight into metabolic risk stratification in women with PCOS.

Upon evaluation of the data, our study demonstrates significant alignment with the existing literature on higher CLU levels in the PCOS cohort and their predictive ability for metabolic syndrome. However, while Asar et al. indicated higher CLU levels in women with PCOS accompanied by metabolic syndrome, their findings were published in an abstract format, thus providing limited data and lacking comprehensive details on the metabolic syndrome diagnosis, ROC analysis, or multivariate regression outcomes [21]. Consequently, the autonomous function of CLU in forecasting metabolic syndrome was not assessed in this investigation. Conversely, our study illustrated CLU’s capacity to differentiate the metabolic phenotype in PCOS by ROC analysis; additionally, backward LR multivariate regression analysis indicated that CLU serves as an independent predictor of metabolic syndrome. From this viewpoint, we assert that our study, characterized by a more thorough analytical approach and rigorous statistical modeling, illustrates that CLUg may serve as a biomarker for metabolic risk classification in PCOS, thus significantly contributing to the current literature. However, a limitation of this study is the lack of additional inflammatory biomarkers, such as high-sensitivity C-reactive protein or retinol-binding protein 4, which were not routinely available during the study period. Inclusion of these markers in future studies may provide further insight into the metabolic phenotype of PCOS.

## 5. Conclusions

The results of our investigation indicate that serum CLU levels are markedly higher in women with PCOS than in healthy controls. In the PCOS group, CLU levels were significantly higher in women with metabolic syndrome, and CLU acted as an independent predictor of metabolic syndrome in multivariable analysis. These data indicate that CLU may represent not only the reproductive and endocrine severity of PCOS but also its cardiometabolic burden, hence supporting its potential function as a supplementary biomarker in clinical risk assessment. Extensive multicenter studies are necessary to confirm the diagnostic and prognostic value of CLU in PCOS and to ascertain its efficacy in accurately identifying high-risk metabolic abnormalities. Subsequent longitudinal and interventional studies should assess if CLU levels fluctuate in response to various treatment modalities and if such fluctuations possess therapeutic significance.

## Figures and Tables

**Table 1:** Demographic characteristics and findings between the PCOS and control groups.

Variables	Control (*n*:40)	PCOS (*n*:40)	*p*-Value
**Age (years)**	24.8 ± 3.0	23.8 ± 3.5	0.164 ^a^
**Gravidity (*n*)**	1 (0–1)	0 (0–0)	**0.002 ^b^**
**Parity (*n*)**	1(0–1)	0 (0–0)	**0.003 ^b^**
**BMI (kg/m^2^)**	24.5 (22.0–28.8)	25.3 (24.2–28.0)	0.079 ^b^
**Follicle count (*n*)**	5 (0–8)	13 (12–15)	**<0.001 ^b^**
**Number of menstrual cycles per year (*n*)**	12 (11–12)	7 (6–8)	**<0.001 ^b^**
**Ferriman–Gallwey score (*n*)**	5 (4–8)	10 (9–12)	**<0.001 ^b^**
**Polycystic morphology on ultrasonography (*n*)**	2 (5.0%)	38 (95.0%)	**<0.001 ^c^**
**Oligo-Anovulation (*n*)**	3 (7.5%)	37 (92.5%)	**<0.001 ^c^**

^a^ Independent-sample *t*-test was used for comparisons between groups. Data are presented as mean ± standard deviation. ^b^ The Mann–Whitney U test was used for comparisons between groups. Data are presented as median (interquartile range). ^c^ Categorical variables were compared using the chi-square or Fisher’s exact test, as appropriate. Results are shown as *n* (%). Bold values indicate statistical significance (*p* < 0.05). Abbreviations: PCOS, polycystic ovary syndrome; BMI, body mass index.

**Table 2:** Hematological, biochemical, and coagulation parameters between the PCOS and control groups.

Variables	Control (*n*:40)	PCOS (*n*:40)	*p*-Value
**Fasting glucose (mg/dL)**	86 (82–94)	89 (84–98)	**0.032 ^b^**
**Fasting insulin (IU)**	10.31 ± 3.86	14.40 ± 5.88	**<0.001 ^a^**
**HOMA-IR score**	2.28 (1.59–3.20)	2.63 (2.02–4.18)	**0.002 ^b^**
**HDL (mg/dL)**	56.0 (45.50–65.0)	50.0 (43.50–55.5)	0.057 ^b^
**LDL (mg/dL)**	95.0 (80.0–118.75)	103.5 (92.75–143.25)	0.111 ^b^
**FSH (mIU/mL)**	5.35 (4.60–6.50)	5.36 (4.35–5.75)	0.256 ^b^
**LH (IU/L)**	5.80 (4.34–6.45)	7.75 (5.96–9.70)	**<0.001 ^b^**
**LH/FSH ratio**	0.948 (0.803–1.214)	1.436 (1.074–2.205)	**<0.001 ^b^**
**E2 (pg/mL)**	41.0 (30.9–59.3)	33.0 (23.3–42.2)	0.070 ^b^
**TSH (mIU/L)**	1.6 (1.2–2.1)	1.5 (0.9–2.7)	0.613 ^b^
**17-OH-P (ng/mL)**	0.36 (0.20–0.63)	0.3 (0.20–0.51)	0.714 ^b^
**Prolactin (µg/L)**	13.9 (9.3–18.8)	14.5 (10.7–24.3)	0.240 ^b^
**Total testosterone (ng/dL)**	23.00 (19.00–30.00)	38.5 (26.5–54.0)	**<0.001 ^b^**
**DHEA-S (µg/dL)**	200 ± 83.5	238 ± 96.9	0.060 ^a^
**SHBG (µL)**	70.5 (39.5–102.8)	46.5 (34.5–65.5)	0.067 ^b^
**AMH (ng/mL)**	2.7 (1.7–3.9)	6.8 (4.9–9.1)	**<0.001 ^b^**
**FAI**	3.72 (2.01–7.20)	7.17 (4.02–14.17)	**0.002 ^b^**
**TG**	114.7 ± 22.7	134.2 ± 24.6	**<0.001**
**Systolic Blood Pressure**	113 (105–121)	132 (125–137)	**<0.001**
**Diastolic Blood Pressure**	75 ± 6	78 ± 8	0.053
**Clusterin (ng/mL)**	21.39 ± 1.97	24.84 ± 2.81	**<0.001 ^a^**

^a^ Independent-sample *t*-test was used for comparisons between groups. Data are presented as mean ± standard deviation. ^b^ The Mann–Whitney U test was used for comparisons between groups. Data are presented as median (interquartile range). Bold values indicate statistical significance (*p* < 0.05). Abbreviations: PCOS, polycystic ovary syndrome; HOMA-IR, Homeostatic Model Assessment of Insulin Resistance; HDL, high-density lipoprotein; LDL, low-density lipoprotein; FSH, follicle-stimulating hormone; LH, luteinizing hormone; E2, estradiol; TSH, thyroid-stimulating hormone; 17-OH-P, 17-hydroxyprogesterone; DHEA-S, dehydroepiandrosterone sulfate; SHBG, sex hormone-binding globulin; AMH, anti-Müllerian hormone; FAI, free androgen index.

**Table 3:** The correlations between clusterin and other variables in the PCOS cohort.

Variables	Clusterin	
	*r*	*p*-Value *
**BMI**	0.236	0.143 ^a^
**Follicle count (*n*)**	−0.125	0.442 ^b^
**Number of menstrual cycles per year (*n*)**	0.134	0.409 ^b^
**Ferriman Gallwey score (*n*)**	0.456	0.003 ^a^
**Fasting Plasma Glucose**	0.457	0.003 ^a^
**Fasting Insulin**	0.619	<0.001 ^b^
**HOMA-IR score**	0.597	<0.001 ^a^
**HDL**	−0.419	**0.007** ^a^
**LDL**	−0.051	0.754 ^a^
**E2**	−0.179	0.270 ^a^
**FSH**	−0.033	0.842 ^b^
**LH**	0.168	0.300 ^a^
**TSH**	0.089	0.585 ^a^
**LH/FSH Ratio**	0.130	0.422 ^a^
**Total testosterone (ng/dL)**	0.133	0.414 ^a^
**FAI**	0.079	0.628 ^a^
**TG**	0.346	0.029 ^b^
**Systolic Blood Pressure**	0.115	0.479 ^a^
**Diastolic Blood Pressure**	0.191	0.238 ^b^
**AMH (ng/mL)**	−0.136	0.403 ^a^

^a^ Spearman’s correlation analysis was used to assess associations between continuous variables. ^b^ Pearson correlation analysis was used to assess associations between continuous variables. * Bold values indicate statistical significance (*p* < 0.05). Abbreviations: PCOS, polycystic ovary syndrome; HOMA-IR, Homeostatic Model Assessment of Insulin Resistance; FSH, follicle-stimulating hormone; LH, luteinizing hormone; AMH, anti-Müllerian hormone.

**Table 4:** Comparison of clinical, hormonal, and metabolic parameters between PCOS patients with and without metabolic syndrome.

Variables	MS (+) (*n* = 17)	Non-MS (*n* = 23)	*p*-Value
**Age (years)**	24.6 ± 3.9	23.2 ± 3.1	0.227 ^a^
**Follicle count (*n*)**	13 ± 3	14 ± 2	0.404 ^a^
**Number of menstrual cycles per year (*n*)**	7 ± 1	7 ± 2	0.059 ^a^
**Ferriman–Gallwey score (*n*)**	12 (9–14)	10 (9–10)	0.017 ^b^
**HOMA-IR score**	4.50 ± 2.02	2.64 ± 1.06	0.002 ^a^
**FSH (mIU/mL)**	5.00 ± 0.84	5.44 ± 1.17	0.198 ^a^
**LH (IU/L)**	8.74 (6.20–11.10)	7.20 (5.60–9.10)	0.448 ^b^
**LH/FSH ratio**	1.827 ± 0.731	1.523 ± 0.590	0.154 ^a^
**E2 (pg/mL)**	31.0 (26.3–36.5)	34.0 (32.0–49.0)	0.156 ^b^
**TSH (mIU/L)**	1.25 (0.90–2.70)	1.60 (1.00–2.50)	0.645 ^b^
**17-OH-P (ng/mL)**	0.430 (0.200–0.580)	0.300 (0.200–0.510)	0.665 ^b^
**Prolactin (µg/L)**	13.3 (8.7–20.0)	16.9 (11.0–25.0)	0.432 ^b^
**Total testosterone (ng/dL)**	40 (31–48)	33 (21–56)	0.481 ^b^
**DHEA-S (µg/dL)**	255 ± 104	226 ± 92	0.353 ^a^
**SHBG (µL)**	40.2 (30.4–62.8)	48.0 (41.2–81.0)	0.242 ^b^
**AMH (ng/mL)**	6.8 (5.6–8.8)	6.6 (4.9–10.2)	0.892 ^b^
**FAI**	7.96 (6.03–16.86)	7.16 (2.27–12.80)	0.221 ^b^
**Clusterin (ng/mL)**	27.61 (25.66–28.22)	23.48 (21.86–24.49)	**<0.001 ^b^**

^a^ Independent-sample *t*-test was used for comparisons between groups. Data are presented as mean ± standard deviation. ^b^ The Mann–Whitney U test was used for comparisons between groups. Data are presented as median (interquartile range). Bold values indicate statistical significance (*p* < 0.05). Abbreviations: PCOS, polycystic ovary syndrome; MS, metabolic syndrome; HOMA-IR, Homeostatic Model Assessment of Insulin Resistance; HDL, high-density lipoprotein; LDL, low-density lipoprotein; FSH, follicle-stimulating hormone; LH, luteinizing hormone; E2, estradiol; TSH, thyroid-stimulating hormone; 17-OH-P, 17-hydroxyprogesterone; DHEA-S, dehydroepiandrosterone sulfate; SHBG, sex hormone-binding globulin; AMH, anti-Müllerian hormone; FAI, free androgen index.

**Table 5:** Univariable and multivariable logistic regression analyses for predictors of PCOS in the entire cohort and predictors of metabolic syndrome within the PCOS group.

Variables	Univariable			Multivariable		
	OR	%95 CI	*p*-Value	aOR	%95 CI	*p*-Value
**For PCOS in the Entire Cohort**						
**Age**	0.907	0.790–1.041	0.163	-	-	**-**
**BMI**	1.082	0.963–1.215	0.187	-	-	**-**
**HOMA-IR**	1.863	1.247–2.783	**0.002**	-	-	**-**
**LH/FSH ratio**	16.258	3.999–66.094	**<0.001**	7.810	1.436–42.476	**0.017**
**Total Testosterone (ng/dL)**	1.076	1.034–1.121	**<0.001**	-	-	**-**
**Clusterin (ng/mL)**	1.807	1.371–2.380	**<0.001**	1.853	1.252–2.743	**0.002**
**AMH (ng/mL)**	1.561	1.268–1.922	**<0.001**	1.564	1.202–2.036	**<0.001**
**For metabolic syndrome in the PCOS Cohort**						
**Age**	1.124	0.932–1.335	**0.222**	1.328	1.001–1.762	**0.049**
**Ferriman–Gallwey score**	1.769	1.176–2.663	**0.006**	1.816	1.073–3.075	**0.026**
**HOMA-IR**	2.190	1.276–3.758	**0.004**	-	-	**-**
**LH/FSH ratio**	2.065	0.762–5.598	**0.154**	-	-	**-**
**FAI**	1.067	0.976–1.167	**0.153**	-	-	**-**
**Clusterin (ng/mL)**	1.631	1.193–2.228	**0.002**	1.577	1.099–2.263	**0.013**

Note: Multivariable analyses were performed using the backward likelihood ratio (LR) logistic regression method. Bold values indicate statistical significance (*p* < 0.05). Abbreviations: LR, logistic regression; OR, odds ratio; aOR, adjusted odds ratio; CI, confidence interval; BMI, body mass index; HOMA-IR, Homeostatic Model Assessment of Insulin Resistance; FSH, follicle-stimulating hormone; LH, luteinizing hormone; AMH, anti-Müllerian hormone; FAI, free androgen index.

**Table 6:** Diagnostic performance of biomarkers for PCOS and metabolic syndrome.

Variables	Cut-Off Value	AUC	95% CI	Sensitivity (%)	Specificity (%)	*p*-Value
**Predicting PCOS in the Entire Cohort**						
**Clusterin (ng/mL)**	≥22.68	0.834	0.746–0.921	78	83	**<0.001**
**LH/FSH Ratio**	≥1.655	0.798	0.702–0.894	48	100	**<0.001**
**AMH (ng/mL)**	≥4.82	0.841	0.752–0.929	78	85	**<0.001**
**Predicting Metabolic Syndrome in PCOS Cohort**						
**Clusterin (ng/mL)**	≥27.30	0.804	0.659–0.950	65	91	**<0.001**

Abbreviations: AUC, area under the curve; CI, confidence interval; PCOS, polycystic ovary syndrome; FSH, follicle-stimulating hormone; LH, luteinizing hormone; AMH, anti-Müllerian hormone.

## Data Availability

Due to hospital policies, patient data and study materials cannot be shared. However, the data are available from the corresponding author upon reasonable request.

## Abbreviations

AMH	Anti-Müllerian Hormone
aOR	Adjusted Odds Ratio
AUC	Area Under the Curve
BMI	Body Mass Index
CI	Confidence Interval
DHEA-S	Dehydroepiandrosterone Sulfate
E2	Estradiol
FAI	Free Androgen Index
FSH	Follicle-Stimulating Hormone
HDL	High-Density Lipoprotein
HOMA-IR	Homeostatic Model Assessment of Insulin Resistance
LDL	Low-Density Lipoprotein
LH	Luteinizing Hormone
NLR	Neutrophil-to-Lymphocyte Ratio
OR	Odds Ratio
OS	Oxidative Stress
PCOS	Polycystic Ovary Syndrome
PDW	Platelet Distribution Width
PLR	Platelet-to-Lymphocyte Ratio
ROC	Receiver Operating Characteristic
SHBG	Sex Hormone-Binding Globulin
TSH	Thyroid-Stimulating Hormone
WBC	White Blood Cell
17-OH-P	17-Hydroxyprogesterone
